# Genetic Polymorphism of *ADORA2A* Is Associated With the Risk of Epilepsy and Predisposition to Neurologic Comorbidity in Chinese Southern Children

**DOI:** 10.3389/fnins.2020.590605

**Published:** 2020-11-11

**Authors:** Xiaomei Fan, Yuna Chen, Wenzhou Li, Hanbin Xia, Bin Liu, Huijuan Guo, Yanxia Yang, Chenshu Xu, Shaojie Xie, Xueqing Xu

**Affiliations:** ^1^Department of Pharmacy, Shenzhen Baoan Women’s and Children’s Hospital, Jinan University, Shenzhen, China; ^2^School of Pharmaceutical Sciences, Health Science Center, Shenzhen University, Shenzhen, China

**Keywords:** *ADORA2A*, polymorphism, childhood epilepsy, neurologic comorbidity, developmental comorbidity, target-pathway analysis

## Abstract

Epilepsy, a common disorder of the brain, exhibits a high morbidity rate in children. Childhood epilepsy (CE) is frequently comorbid with neurologic and developmental disorders, sharing underlying genetic factors. This study aimed to investigate the impact of *ADORA2A*, *BDNF*, and *NTRK2* gene polymorphisms on the risk of childhood epilepsy and their associations with predisposition to epileptic comorbidities. A total of 444 children were enrolled in this study, and three single nucleotide polymorphisms, including *ADORA2A* rs2298383, *BDNF* rs6265, and *NTRK2* rs1778929, were genotyped. The frequency distribution of genotypes was compared not only between CE patients and healthy children but also between CE patients with and without comorbidities. The results indicated that the carriers of *ADORA2A* rs2298383 TT genotype tended to have a lower risk of epilepsy (OR = 0.48, 95% CI = 0.30–0.76), while the CT genotype was related to a higher risk (OR = 1.56, 95% CI = 1.06–2.27). The *ADORA2A* rs2298383 CC genotype predisposed CE patients to comorbid neurologic disorders (OR = 2.76, 95% CI = 1.31–5.80). Genetic variations in *BDNF* rs6265 and *NTRK2* rs1778929 had no significant association with CE and its comorbidities. Fourteen ADORA2A target genes related to epilepsy were identified by the protein–protein interaction analysis, which were mainly involved in the biological processes of “negative regulation of neuron death” and “purine nucleoside biosynthetic process” through the gene functional enrichment analysis. Our study revealed that the genetic polymorphism of *ADORA2A* rs2298383 was associated with CE risk and predisposition to neurologic comorbidity in children with epilepsy, and the involved mechanism might be related to the regulation of neuron death and purine nucleoside biosynthesis.

## Introduction

As a brain disorder, epilepsy is characterized by an enduring predisposition to generate epileptic seizures with neurobiological, cognitive, psychological, and social consequences (Fisher et al., 2005, 2014). With 65 million people suffering worldwide, epilepsy is regarded as the most common, chronic, and serious neurologic disease, which occurs at any age but is highly prevalent in children and the elderly (Thurman et al., 2011; Moshé et al., 2015). Children with epilepsy are at an elevated risk of a wide range of neurologic, developmental, and psychiatric disorders, which precede, co-occur with, or follow the diagnosis of epilepsy. Nearly 80% of children with epilepsy have at least one comorbid disorder. The high overall frequency of comorbid diseases is observed in children with all types of epilepsy, even for presumably uncomplicated epilepsies (Aaberg et al., 2016). The comorbid disorders may share common causes or risk factors with epilepsy or are even the actual cause of epilepsy (Aaberg et al., 2016). This indicates an underlying shared etiology between epilepsy and the comorbidities, which is multifactorial and involves genetic contribution (Seidenberg et al., 2009). At present, the growing prevalence of genetic testing has permitted to identify the genes involved in epilepsy predisposition, development, and subtype classification. Recent studies have shown numerous genes associated with epilepsy (Wang et al., 2017), some of which are also associated with neurologic and developmental disorders (Fassio et al., 2018; Mulhern et al., 2018; Kanca et al., 2019). However, the genetic basis of epilepsy remains largely unknown, and further studies discovering reliable markers in epilepsy genomics are still needed.

Adenosine is an essential neuromodulator that plays an important role in various pathophysiological conditions through affecting sleep regulation, pain, blood pressure, neuronal survival, and psychomotor behavior (Fredholm et al., 2011). Numerous evidences have revealed the complex roles of adenosine in the brain, deriving from the diversity of receptor subtypes. The adenosine A(2A) receptor (ADORA2A) abundantly expressed in the brain has emerged as a key therapeutic target for Parkinson’s disease and potentially other neuropsychiatric disorders (Fernández-Dueñas et al., 2019; Oliveira et al., 2019). The *ADORA2A* gene encodes a member of the guanine nucleotide-binding protein (G protein)-coupled receptor (GPCR) superfamily and uses adenosine as the preferred endogenous agonist to increase intracellular cAMP levels. ADORA2A competes with adenosine A1 receptor (ADORA1) in various neural functions and enhances excitatory neurotransmitter release for synaptic transmission (Fredholm et al., 2005). The *Adora2a* knockout mice have been reported to show a reduction of ethanol-induced seizures (El Yacoubi et al., 2001). Furthermore, the AA diplotype of *ADORA2A* was found to predispose Japanese children to acute encephalopathy with biphasic seizures and late reduced diffusion by altering the intracellular adenosine/cAMP signal cascade (Shinohara et al., 2013). These studies suggest that the *ADORA2A* gene may be related to epilepsy. However, the role and the underlying mechanisms of ADORA2A in epilepsy remain unclear and need to be elucidated.

Additionally, a cross-talk between ADORA2A and neurotrophin brain-derived neurotrophic factor (BDNF) exists. BDNF, essential in the regulation of neuronal survival and differentiation, synaptic transmission, and plasticity, plays a pivotal role in the pathogenesis of epilepsy and central comorbid conditions associated with epilepsy (Kanemoto et al., 2003). BDNF activates neurotrophic receptor tyrosine kinase 2 (NTRK2) to elicit its effects on neural activity and cognition (Nair and Vaidya, 2006). ADORA2A is not only involved in the transactivation of BDNF receptor NTRK2 but also modulates BDNF levels and its effect on synaptic transmission (Sharma et al., 2019). Activation of adenosine receptor A2A-R (encoded by ADORA2A) as well as NTRK2 receptors and BDNF is associated with neuroprotective signaling (Queiroga et al., 2016; Pradhan et al., 2019). The genetic associations of *BDNF* and *NTRK2* with epilepsy have been studied, while their roles in epilepsy remain controversial and need to be verified.

In our study, we chose three representative single nucleotide polymorphisms (SNPs) of *ADORA2A*, *BDNF*, and *NTRK2* genes(Supplementary Table S1) to investigate the association of their genetic polymorphism with the risk of childhood epilepsy (CE) and predisposition of this disease to comorbid neurologic and developmental disorders in Chinese southern children. The bioinformatics analysis was used to explore the target pathway/function network of the potential susceptible genes in epilepsy for predicting their mechanisms.

## Patients and Methods

### Study Participants

A total of 200 patients with CE and 244 healthy children were recruited at Baoan Women’s and Children’s Health Hospital of Jinan University in China between October 2016 and December 2019. All the CE patients were diagnosed and interviewed by two independent neurologic clinicians according to the latest ILAE Commission’s classification criterion (Berg et al., 2010; Scheffer et al., 2017). Children aged < 16 years who were diagnosed with localized or generalized epilepsy, epileptic syndrome, or other symptomatic generalized epilepsy were included in our study. The diagnosis of epilepsy was restricted to the period of 1 month after birth for patients to avoid misclassifying neonatal convulsions as epilepsy. Patients without a clear diagnosis of epilepsy were excluded. The exclusion criteria included non-reliable seizure frequency, a history of pseudoseizures, or the presence of any diseases that may aggravate epilepsy, such as hepatic or renal diseases. Only an individual without any nervous system diseases, developmental retardation, or family history of epilepsy was eligible for inclusion in the control group. The procedures of this study were approved by Baoan Women’s and Children’s Hospital Ethics Committee. Written informed consent was obtained from all guardians of the participants in the study. The clinical information of patients including sex, age, disease diagnosis, family history of seizure and epilepsy, perinatal and neonatal complications, and comorbid neurologic and developmental conditions were collected. Children missing the above-mentioned clinical data were excluded from this study. The participants with CE (*n* = 200) were further grouped into four categories based on clinical diagnosis: CE only (*n* = 105), CE with neurologic comorbidity (NC) (*n* = 49), CE with developmental comorbidity (DC) (*n* = 82), and CE with both neurologic and developmental comorbidities (NC/DC) (*n* = 36).

The neurologic comorbidities included cerebral palsy, hydrocephalus, cerebral injury, encephalopathy, encephalitis, meningitis, hydrocephalus, microcephaly, schizencephaly, pachygyria, and intracerebral hemorrhage. Developmental disorders were characterized by developmental impairment in reciprocal social interaction and communication skills and the presence of stereotyped behavior, interests, and activities. The qualitative impairments that defined the above-mentioned conditions were distinctly deviant relative to the individual’s developmental level or mental age. The developmental comorbidities in this study included intellectual and developmental disabilities, ADHD, early language disorder, autism, delayed psychomotor development, and mental and motor retardation. For CE with NC/DC group, the patients who presented CE and neurologic or developmental disorders simultaneously were included.

### SNP Selection and Genotyping

Genomic DNA was extracted from 1.5 ml of peripheral blood samples collected in ethylenediaminetetraacetic acid anticoagulation tubes according to previously reported methods (Huang et al., 2015). Polymerase chain reaction (PCR) was amplified under standard PCR conditions according to the manufacturer’s instructions. Briefly, 10 ng of template DNA and 0.5 μM of primer were contained in a PCR primer mixture. The protocol of PCR reaction consisted of initial denaturation at 95°C for 2 min, followed by 45 cycles of denaturation at 95°C for 30 s, annealing at 56°C for 30 s, and extension at 72°C for 1 min, and followed by a final extension of 72°C for 5 min. After the PCR reaction, the PCR products were purified with shrimp alkaline phosphatase treatment under the guidance of the manufacturer’s recommendation. Genotyping of all polymorphisms was carried out with the Sequenom MassArray platform (Agena Bioscience, San Diego, CA, United States) and iPLEX Gold Assay. The MassArray system includes the MassArray Analyzer mass spectrometer and integrated data analysis software. The genotyping primers of *ADORA2A* rs2298383, *BDNF* rs6265, and *NTRK2* rs1778929 were specifically designed in this study (forward primer sequence: 5′-ACGTTGGATGGAGGGATAGGAAATTGC CAG-3′ and reverse primer sequence: 5′-ACGTTGGATGAG AGTGACAGGAAGGTCAGA-3′ for *ADORA2A* rs2298383; forward primer sequence: 5′-ACGTTGGATGCTTCATTGGG CCGAACTTTC-3′ and reverse primer sequence: 5′-ACGTT GGATGGCTTGACATCATTGGCTGAC-3′ for *BDNF* rs6265; and forward primer sequence: 5′-ACGTTGGATGG AAAAGTAAGCTTTTGGGTG-3′ and reverse primer sequence: 5′-ACGTTGGATGTTTTTTCTTTGCTGGGAGGG-3′ for *NTRK2* rs1778929). The MassArray Typer 4.0 software was used for data acquisition and analyses.

### Analysis of Protein–Protein Interaction Network

Protein–protein interaction (PPI) elucidates the function of regulation among proteins and thus is helpful to studying protein function (Vella et al., 2017). To further analyze the interaction network of ADORA2A, STRING database v11.0^1^, a public database containing interactions between known and predicted proteins (Szklarczyk et al., 2018), was used to assemble PPI networks. The PPI networks were clustered by using k-means clustering method. “Text-mining,” “Experiments,” “Databases,” “Co-expression,” “Neighborhood,” “Gene Fusion,” and “Co-occurrence” were used as prediction methods. Only *Homo sapiens* proteins linked to ADORA2A were selected for PPI analysis. The number of ADORA2A-interacted proteins in the PPI network was restricted by setting an interaction score value and the number of interactors. In this study, the minimum required interaction score chose default medium confidence (0.400) as cutoff criteria. The maximum number of interactors selected was not more than 20 (1st shell not more than 20). The PPI network was further visualized by Cytoscape v3.6.0.

### Potential Epilepsy-Associated Targets of *ADORA2A*

The epilepsy-associated genes were obtained from DisGeNET^2^ and GeneCards database^3^. DisGeNET, a discovery platform for the dynamical exploration of human diseases and their associated genes, is considered as a good tool for investigating the molecular mechanisms underlying diseases of genetic origin (Pinero et al., 2015). GeneCards provides a web-based card for each of the tens of thousands of human gene entries through sharing gene-centric information, automatically mining and integrating from myriad data sources. We searched “epilepsy” in DisGeNET and GeneCards. The epilepsy-associated genes retrieved from DisGeNET were further filtered by the number of Pubmed IDs (PMIDs) > 0. The relevance score ≥ 1 was used in GeneCards. All the epilepsy-associated genes from the above-mentioned two databases were intersected with the genes related to ADORA2A from PPI analysis in section “Analysis of Protein–Protein Interaction Network.” The potential targets of *ADORA2A* on epilepsy were obtained by using a Venn diagram package.

### Functional Enrichment Analysis

To further analyze the signaling pathways and the functions of potential target genes, pathway and process enrichment analysis for common genes of *ADORA2A* on epilepsy was performed using Metascape bioinformatics tool. Metascape^4^ is a web-based portal designed to provide a comprehensive gene list annotation and analysis resource (Zhou et al., 2019). Metascape analysis is performed with four gene ontology (GO) sources: GO Biological Process, Reactome Gene Sets, Kyoto Encyclopedia of Genes and Genomes (KEGG) pathway, and the Comprehensive Resource of Mammalian protein complexes. All statistically enriched terms, including GO/KEGG terms and canonical pathways, shall mark gene sets that were identified. All statistically enriched terms were identified, and accumulative hypergeometric *p*-values and enrichment factors (the ratio between the observed counts and the counts expected by chance) were calculated and used for filtering. Terms with a *p*-value < 0.01, a minimum count of 3, and an enrichment factor > 1.5 were collected and grouped into clusters based on their membership similarities. Kappa scores were used as the similarity metric when performing hierarchical clustering on the enriched terms. A subset of representative terms was then selected from the cluster and converted into a network layout. Each term was presented with a circle node, and terms with a similarity score > 0.3 were linked by an edge. The most statistically significant term within a cluster was chosen to represent the cluster. The network was visualized with Cytoscape (v3.6.0).

### Statistical Analysis

The data were expressed as mean ± standard deviation (SD). Differences in the demographic characteristics between the two groups were analyzed by independent-samples *t*-test for continuous variables and chi-square test for categorical data. The chi-square test was used to assess the deviation from Hardy–Weinberg equilibrium. The differences in frequency distributions of genotypes between controls and cases and between CE patients with and without neurologic and developmental comorbidities were assessed by chi-square test or Fisher exact test with Yate corrections. The odds ratio (OR), together with the 95% confidence interval (CI), was calculated for multiple tests in this study. *P*-values less than 0.05 were considered to be statistically significant.

The minimum sample size was calculated with a default alpha value (0.05) and power (0.8) by PASS sample size software (version 2020). The power of our study was computed for verification based on alpha (0.05), calculated effect size, and actual sample size in the present study using R Programming Language (version 3.6.3). Power equal to 0.8 is considered a high probability to obtain a statistically significant result. The results indicated the power > 0.9 based on the frequency distribution of three genotypes *ADORA2A* rs2298383 from controls (*n* = 244) and cases (*n* = 200).

## Results

### Clinical Characteristics of CE Patients

The clinical characteristics of the participants shown in Table 1 indicated that there was no significant difference on the gender of the participants between control and case groups (*p* = 0.47). Among 200 CE patients, the average ages of seizure onset and diagnosis of CE were 2.99 ± 3.09 and 3.43 ± 3.14 years, respectively. Compared with healthy individuals, most of the CE patients experienced febrile seizure (22%), status epilepticus (10%), abnormal pregnancy at perinatal stage (11.5%), abnormal delivery or delayed discharge from the newborn intensive care unit (NICU) (11.5%), and had a family history of epilepsy (15.5%). The main neurologic comorbidities in CE patients were cerebral injury (23.3%), structural brain lesions (21.9%), cerebral palsy (19.2%), and encephalopathy (13.7%). Intellectual disability (30.5%) and dysgenopathy (23.8%) were the two main developmental comorbidities in CE patients. CE patients with the comorbidities tended to be younger at first diagnosis of epilepsy than those without comorbidities, most of whom were diagnosed as epileptic at ages from 2 months to 1 year old. Additionally, the proportion of cases with a history of status epilepticus, abnormal pregnancy, abnormal delivery, or delayed discharge from NICU in the three CE comorbidity groups was significantly higher than that in the CE group, suggesting that these risk factors might be related to the epileptic comorbidities.

**TABLE 1:** Clinical characteristics of children with epilepsy in this study.

Variables	Controls (*n* = 244)	Cases (*n* = 200)	Childhood epilepsy (CE) patients (*n* = 105)	CE patients with NC (*n* = 49)	CE patients with DC (*n* = 82)	CE patients with NC/DC (*n* = 36)	OR (95% CI)^*a*^	*p* value^*a*^	OR (95% CI)^*b*^	*p* value^*b*^	OR (95% CI)^*c*^	*p* value^*c*^
**Age of CE diagnosis (years)**												
1 month–2	34 (13.93%)	96 (48.0%)	38 (36.2%)	31 (63.3%)	52 (63.4%)	25 (69.4%)	0.33	0.0017**	0.33	0.0002***	0.25	0.0005***
2–16	210 (86.07%)	104 (52.0%)	67 (63.8%)	18 (36.7%)	30 (36.6%)	11 (30.6%)	(0.17–0.65)		(0.18–0.59)		(0.12–0.56)	
Mean ± SD	4.57 ± 2.71	3.43 ± 3.11	4.14 ± 3.16	2.04 ± 2.23	2.49 ± 2.90	1.65 ± 1.64	–	<0.0001***	–	0.00025***	–	<0.0001***
**Gender**												
Male	132 (54.10%)^*d*^	115 (57.5%)	61 (58.1%)	28 (57.1%)	45 (54.9%)	19 (52.8%)	1.04	0.91	1.14	0.66	1.24	0.58
Female	112 (45.90%)^*d*^	85 (42.5%)	44 (41.9%)	21 (42.9%)	37 (45.1%)	17 (47.2%)	(0.52–2.03)		(0.64–2.03)		(0.57–2.65)	
**Febrile seizure**												
Yes	0 (0%)	44 (22.0%)	28 (26.7%)	8 (16.3%)	13 (15.8%)	5 (13.9%)	1.86	0.16	1.93	0.076	2.25	0.12
No	224 (100%)	156 (78.0%)	77 (73.3%)	41 (83.7%)	69 (84.2%)	31 (86.1%)	(0.78–4.27)		(0.94–3.87)		(0.81–5.75)	
**History of status epilepticus**												
Yes	0 (0%)	20 (10.0%)	2 (1.9%)	15 (30.6%)	11 (13.4%)	8 (22.2%)	0.044	<0.0001***	0.13	0.0054**	0.068	0.0002***
No	224 (100%)	180 (90.0%)	103 (98.1%)	34 (69.4%)	71 (86.6%)	28 (77.8%)	(0.0098–0.18)		(0.027–0.55)		(0.014–0.31)	
**History of abnormal pregnancy**												
Yes	0 (0%)	23 (11.5%)	3 (2.9%)	12 (24.5%)	19 (23.2%)	11 (30.6%)	0.091	<0.0001***	0.098	<0.0001***	0.044	<0.0001***
No	224 (100%)	177 (88.5%)	102 (97.1%)	37 (75.5%)	63 (76.8%)	25 (69.4%)	(0.027–0.32)		(0.030–0.32)		(0.0096–0.21)	
**Abnormal delivery or delayed discharge from NICU**												
Yes	0 (0%)	23 (11.5%)	3 (2.9%)	11 (22.5%)	19 (23.2%)	10 (27.8%)	0.10	0.0003***	0.098	<0.0001***	0.076	<0.0001***
No	224 (100%)	177 (88.5%)	102 (97.1%)	38 (77.5%)	63 (76.8%)	26 (72.2%)	(0.030–0.37)		(0.030–0.32)		(0.022–0.30)	
**Family history of epilepsy**												
Yes	0 (0%)	31 (15.5%)	19 (18.1%)	5 (10.2%)	9 (11.0%)	2 (5.6%)	1.94	0.21	1.79	0.18	3.76	0.068
No	224 (100%)	169 (84.5%)	86 (81.9%)	44 (89.8%)	73 (89.0%)	34 (94.4%)	(0.67–5.01)		(0.75–4.43)		(0.97–16.93)	

^*a*^OR and *p*-value obtained from CE patients group (*n* = 105) *versus* CE patients with NC group (*n* = 49). ^*b*^OR and *p*-value obtained from CE patients group (*n* = 105) *versus* CE patients with DC group (*n* = 82). ^*c*^OR and *p*-value obtained from CE patients group (*n* = 105) *versus* CE patients with NC/DC group (*n* = 36). ^*d*^For gender comparison, *p* = 0.47 and OR = 0.87 (95% CI 0.60–1.26) were obtained from controls (*n* = 244) *versus* cases (*n* = 200). **p < 0.01, ***p < 0.001 *versus* CE patients group.

### The Association of Gene Polymorphism With Childhood Epilepsy Risk

The frequency distribution of *ADORA2A* rs2298383, *BDNF* rs6265, and *NTRK2* rs1778929 genotypes in controls and CE patients is shown in Table 2. For *ADORA2A* rs2298383, the distribution of CC, CT, and TT genotypes was 26.5%, 57%, and 16.5%, respectively, in the case group compared to 25%, 45.9%, and 29.1% in the control group (*p* = 0.0055). T allele was less frequent in CE patients than in healthy children (45 and 52%, respectively) as evidenced by the allele analysis. Carriers of at least one polymorphic *ADORA2A* rs2298383 C allele tended to have a higher epilepsy risk for children (OR = 2.08, 95% CI = 1.31–3.30), while TT genotype was associated with a lower risk (OR = 0.48, 95% CI = 0.30–0.76; *p* = 0.0016). Homozygous carriers including mutant and wild genotypes (CC and TT) exhibited a lower susceptibility than heterozygous CT (OR = 0.64, 95% CI = 0.44–0.93; *p* = 0.02). For the polymorphism of *BDNF* rs6265 and *NTRK2* rs1778929 gene, there was no significant difference in the frequencies of the genotype distribution between control and CE patients. Carriers of at least one polymorphic *BDNF* rs6265 T allele tended to have a relatively higher epilepsy risk for children (OR = 1.53, 95% CI = 0.99–2.36; *p* = 0.054).

**TABLE 2:** Comparison of *ADORA2A* rs2298383, *BDNF* rs6265, and *NTRK2* rs1778929 diplotype distribution between healthy children and childhood epilepsy (CE) patients.

Single nucleotide polymorphism	Genetic model	Diplotype	Cases (*n* = 200)	Controls (*n* = 244)	OR (95% CI)	*p-*value
*ADORA2A* rs2298383	Allele contrast	C *vs.* T	220 (55.0%)/180 (45.0%)	234 (48.0%)/254 (52.0%)	1.00, 1.33 (1.02–1.73)	0.037*
	Codominant	CC *vs.* CT *vs.* TT	53 (26.5%)/114 (57.0%)/33 (16.5%)	61 (25.0%)/112 (45.9%)/71 (29.1%)	1.00, 0.85 (0.54–1.34) 1.87 (1.08–3.25)	0.0055**
	Dominant	CC *vs.* CT + TT	53 (26.5%)/147 (73.5%)	61 (25.0%)/183 (75.0%)	1.00, 1.08 (0.71–1.66)	0.72
	Recessive	CC + CT *vs.* TT	167 (83.5%)/33 (16.5%)	173 (70.9%)/71 (29.1%)	1.00, 2.08 (1.31–3.30)	0.0016**
	Overdominant	CC + TT *vs.* CT	86 (43.0%)/114 (57.0%)	132 (54.1%)/112 (45.9%)	1.00, 0.64 (0.44–0.93)	0.02*
	Log-additive	CC *vs.* TT	53 (26.5%)/33 (16.5%)	61 (25.0%)/71 (29.1%)	1.00, 1.34 (1.02–1.75)	0.034*
*BDNF* rs6265	Allele	T *vs.* C	213 (53.3%)/187 (46.7%)	236 (48.4%)/252 (51.6%)	1.00, 1.22 (0.93–1.59)	0.15
	Codominant	TT *vs.* CT *vs.* CC	56 (28.0%)/101 (50.5%)/43 (21.5%)	64 (26.2%)/108 (44.3%)/72 (29.5%)	1.00, 0.94 (0.60–1.47) 1.47 (0.87–2.47)	0.15
	Dominant	TT *vs.* CT + CC	56 (28.0%)/144 (72.0%)	64 (26.2%)/180 (73.8%)	1.00, 1.09 (0.72–1.67)	0.68
	Recessive	TT + CT *vs.* CC	157 (78.5%)/43 (21.5%)	172 (70.5%)/72 (29.5%)	1.00, 1.53 (0.99–2.36)	0.054
	Overdominant	TT + CC *vs.* CT	99 (49.5%)/101 (50.5%)	136 (55.7%)/108 (44.3%)	1.00, 0.78 (0.53–1.13)	0.19
	Log-additive	TT *vs.* CC	56 (28.0%)/43 (21.5%)	64 (26.2%)/72 (29.5%)	1.00, 1.20 (0.93–1.56)	0.16
*NTRK2* rs1778929	Allele	C *vs.* T	237 (59.2%)/163 (40.8%)	276 (56.6%)/212 (43.4%)	1.00, 1.12 (0.85–1.47)	0.42
	Codominant	CC *vs.* CT *vs.* TT	65 (32.5%)/107 (53.5%)/28 (14.0%)	74 (30.3%)/128 (52.5%)/42 (17.2%)	1.00, 1.05 (0.69–1.60) 1.32 (0.74-2.36)	0.63
	Dominant	CC *vs.* CT + TT	65 (32.5%)/135 (67.5%)	74 (30.3%)/170 (69.7%)	1.00, 1.11 (0.74–1.65)	0.62
	Recessive	CC + CT *vs.* TT	172 (86.0%)/28 (14.0%)	202 (82.8%)/42 (17.2%)	1.00, 1.28 (0.76–2.15)	0.35
	Overdominant	CC + TT *vs.* CT	93 (46.5%)/107 (53.5%)	116 (47.5%)/128 (52.5%)	1.00, 0.96 (0.66–1.39)	0.83
	Log-additive	CC *vs.* TT	65 (32.5%)/28 (14.0%)	74 (30.3%)/42 (17.2%)	1.00, 1.13 (0.85–1.49)	0.40

*p < 0.05, **p < 0.01 *versus* control group.

### The Association of Gene Polymorphism With Predisposition to Neurologic and Developmental Comorbidities in Childhood Epilepsy

The frequency distribution of *ADORA2A* rs2298383, *BDNF* rs6265, and *NTRK2* rs1778929 genotypes in CE patients with and without neurologic and developmental disorders is shown in Table 3. A significant difference in the frequencies of *ADORA2A* rs2298383 genotype distribution was observed between CE patients with and without neurologic disorders. The allele analysis indicated a significant increase in the frequency of C allele for CE patients with NC compared to those without NC (66.3% and 50.5%, respectively, OR = 1.93, 95% CI = 1.17–3.22; *p* = 0.0091). The mutant homozygous TT and heterozygous CT genotypes were associated with a lower risk of neurologic comorbidity for CE patients, while homozygous CC genotype was related to a higher risk (OR = 2.76, 95% CI = 1.31–5.80; *p* = 0.0076). However, the polymorphism of *ADORA2A* rs2298383 was not associated with CE comorbid developmental disorders as evidenced by the lack of significant difference between CE patients with and without DC. In the present study, *BDNF* rs6265 and *NTRK2* rs1778929 gene polymorphisms were not found to be significantly related to the NC and DC for CE patients.

**TABLE 3:** Comparison of *ADORA2A* rs2298383, *BDNF* rs6265, and *NTRK2* rs1778929 diplotype distribution between childhood epilepsy (CE) patients with and without neurologic comorbidities.

Single nucleotide polymorphism		Genetic model		Diplotype		CE patients (*n* = 105)		CE patients with NC (*n* = 49)	CE patients with DC (*n* = 82)		CE patients with NC/DC (*n* = 36)		OR (95% CI)^*a*^		*p*-value^*a*^		OR (95% CI)^*b*^		*p*-value^*b*^		OR (95% CI)^*c*^		*p*-value^*c*^	
*ADORA2A* rs2298383	Allele contrast		C *vs.* T		106 (50.5%)/104 (49.5%)		65 (66.3%)/33 (33.7%)			95 (57.9%)/69 (42.1%)		46 (63.9%)/26 (36.1%)		1.93 (1.17–3.22)		0.0091**		1.35 (0.89–2.02)		0.15		1.74 (1.02–2.99)		0.049*
	Codominant		CC *vs.* CT *vs.* TT		21 (20.0%)/64 (61.0%)/20 (19.0%)		20 (40.8%)/25 (51.0%)/4 (8.2%)			26 (31.7%)/43 (52.4%)/13 (15.9%)		14 (38.9%)/18 (50.0%)/4 (11.1%)		1.00, 2.44 (1.13–5.25) 4.76 (1.38–16.39)		0.014*		1.00, 1.84 (0.92–3.68) 1.90 (0.77–4.71)		0.19		1.00, 2.37 (1.01–5.57) 3.33 (0.94–11.86)		0.076
	Dominant		CC *vs.* CT + TT		21 (20.0%)/84 (80.0%)		20 (40.8%)/29 (59.2%)			26 (31.7%)/56 (68.3%)		14 (38.9%)/22 (61.1%)		1.00, 2.76 (1.31–5.80)		0.0076**		1.00, 1.86 (0.95–3.62)		0.068		1.00, 2.55 (1.12–5.80)		0.028*
	Recessive		CC + CT *vs.* TT		85 (81.0%)/20 (19.0%)		45 (91.8%)/4 (8.2%)			69 (84.1%)/13 (15.9%)		32 (88.9%)/4 (11.1%)		1.00, 2.65 (0.85–8.22)		0.069		1.00 1.25 (0.58–2.69)		0.57		1.00, 1.88 (0.60–5.93)		0.26
	Overdominant		CC + TT *vs.* CT		41 (39.0%)/64 (61.0%)		24 (49.0%)/25 (51.0%)			39 (47.6%)/43 (52.4%)		18 (50.0%)/18 (50.0%)		1.00, 1.50 (0.76–2.97)		0.25		1.00 1.42 (0.79–2.54)		0.24		1.00, 1.56 (0.73–3.34)		0.25
	Log-additive		CC *vs.* TT		21 (20.0%)/20 (19.0%)		20 (40.8%)/4 (8.2%)			26 (31.7%)/13 (15.9%)		14 (38.9%)/4 (11.1%)		1.00, 2.77 (1.28–4.02)		0.0037**		1.43 (0.91–2.25)		0.12		1.00, 1.97 (1.06–3.66)		0.029*
*BDNF* rs6265	Allele contrast		T *vs.* C		112 (53.3%)/98 (46.7%)		58 (59.2%)/40 (40.8%)			87 (53.0%)/77 (47.0%)		43 (59.7%)/29 (40.3%)		1.27 (0.78–2.03)		0.34		0.99 (0.65–1.50)		0.96		1.30 (0.77–2.28)		0.35
	Codominant		TT *vs.* CT *vs.* CC		30 (28.6%)/52 (49.5%)/23 (21.9%)		16 (32.6%)/26 (53.1%)/7 (14.3%)			23 (28.0%)/41 (50.0%)/18 (22.0%)		13 (36.1%)/17 (47.2%)/6 (16.7%)		1.00, 1.07 (0.49–2.30) 1.75 (0.62–4.96)		0.52		1.00 0.97 (0.49–1.92) 0.98 (0.43–2.23)		1.00		1.00, 1.33 (0.57–3.10) 1.66 (0.55–5.04)		0.64
	Dominant		TT *vs.* CC + CT		30 (28.6%)/75 (71.4%)		16 (32.6%)/33 (67.4%)			23 (28.0%)/59 (72.0%)		13 (36.1%)/23 (63.9%)		1.00, 1.21 (0.58–2.52)		0.61		1.00, 0.97 (0.51–1.85)		0.94		1.00, 1.41 (0.63–3.15)		0.40
	Recessive		TT + CT *vs.* CC		82 (78.1%)/23 (21.9%)		42 (85.7%)/7 (14.3%)			64 (78.0%)/18 (22.0%)		30 (83.3%)/6 (16.7%)		1.00, 1.68 (0.67–4.24)		0.26		1.00, 1.00 (0.50–2.00)		0.99		1.00, 1.40 (0.52–3.78)		0.49
	Overdominant		TT + CC *vs.* CT		53 (50.5%)/52 (49.5%)		23 (46.9%)/26 (53.1%)			41 (50.0%)/41 (50.0%)		19 (52.8%)/17 (47.2%)		1.00, 0.87 (0.44–1.71)		0.68		1.00, 0.98 (0.55–1.75)		0.95		1.00, 1.10 (0.51–2.34)		0.81
	Log-additive		CC *vs.* TT		23 (21.9%)/30 (28.6%)		7 (14.3%)/16 (32.6%)			18 (22.0%)/23 (28.0%)		6 (16.7%)/13 (36.1%)		1.28 (0.78–2.09)		0.33		0.99 (0.66–1.49)		0.96		1.00, 1.29 (0.75–2.23)		0.35
*NTRK2* rs1778929	Allele contrast		C *vs.* T		123 (58.6%)/87 (41.4%)		61 (62.2%)/37 (37.8%)			100 (61.0%)/64 (39.0%)		48 (66.7%)/24 (33.3%)		1.17 (0.71–1.90)		0.54		1.10 (0.73–1.67)		0.64		1.41 (0.81–2.50)		0.22
	Codominant		CC *vs.* CT *vs.* TT		34 (32.4%)/55 (52.4%)/16 (15.2%)		18 (36.7%)/25 (51.0%)/6 (12.3%)			27 (32.9%)/46 (56.1%)/9 (11.0%)		15 (41.7%)/18 (50.0%)/3 (8.3%)		1.00, 1.16 (0.55–2.44) 1.41 (0.47–4.23)		0.81		1.00, 0.95 (0.50–1.80) 1.41 (0.54–3.69)		0.68		1.00, 1.35 (0.60–3.02) 2.35 (0.60–9.30)		0.42
	Dominant		CC *vs.* CT + TT		34 (32.4%)/71 (67.6%)		18 (36.7%)/31 (63.3%)			27 (32.9%)/55 (67.1%)		15 (41.7%)/21 (58.3%)		1.00, 1.21 (0.60–2.47)		0.59		1.00, 1.03 (0.55–1.90)		0.94		1.00, 1.49 (0.68–3.25)		0.32
	Recessive		CC + CT *vs.* TT		89 (84.8%)/16 (15.2%)		43 (87.8%)/6 (12.2%)			73 (89.0%)/9 (11.0%)		33 (91.7%)/3 (8.3%)		1.00, 1.29 (0.47–3.52)		0.62		1.00, 1.46 (0.61–3.49)		0.39		1.00, 1.98 (0.54–7.23)		0.27
	Overdominant		CC + TT *vs.* CT		50(47.6%)/55 (52.4%)		24 (49.0%)/25 (51.0%)			36 (43.9%)/46 (56.1%)		18 (50.0%)/18 (50.0%)		1.00, 1.06 (0.54–2.08)		0.87		1.00, 0.86 (0.48–1.54)		0.61		1.00, 1.10 (0.52–2.35)		0.81
	Log-additive		CC *vs.* TT		34 (32.4%)/16 (15.2%)		18 (36.7%)/6 (12.3%)			27 (32.9%)/9 (11.0%)		15 (41.7%)/3 (8.3%)		1.00, 1.18 (0.71–1.98)		0.52		1.00, 1.12 (0.72–1.75)		0.62		1.00, 1.46 (0.81–2.64)		0.20

^*a*^OR and *p*-value obtained from CE patients with NC group (*n* = 49) *versus* CE patients group (*n* = 105). ^*b*^OR and *p*-value obtained from CE patients with DC group (*n* = 82) *versus* CE patients group (*n* = 105). ^*c*^OR and *p*-value obtained from CE patients with NC/DC group (*n* = 36) *versus* CE patients group (*n* = 105). *p < 0.05, **p < 0.01 *versus* CE patients’ group.

Furthermore, the association between three investigated SNPs and the risk of epilepsy with two comorbidities was explored in CE patients with NC/DC. The results showed that the C allele of *ADORA2A* rs2298383 was more frequent in CE comorbid NC/DC patients than that in CE patients without comorbidities compared with T allele as evidenced by the allele analysis (63.9% vs. 50.5% and 36.1% vs. 49.5%, respectively, *p* = 0.049). Homozygous CC genotype was associated with a higher risk of combined neurologic and developmental comorbidities for CE patients than mutant homozygous TT or heterozygous CT genotypes (OR = 2.55, 95% CI = 1.12–5.80; *p* = 0.028).

Additionally, a stratification analysis of *ADORA2A* rs2298383 was further conducted by selected variables in CE patients. As shown in Table 4, a significant difference in the frequency distribution of *ADORA2A* rs2298383 genotypes was observed in CE patients with and without NC or NC/DC in the subgroup of no febrile seizure (*p* = 0.019 and *p* = 0.036, respectively). The CE patients without abnormal delivery or delayed discharge from NICU also exhibited a significant difference in genotype distribution of *ADORA2A* rs2298383 between CE patients with and without NC (*p* = 0.033).

**TABLE 4:** Stratification analysis of *ADORA2A* rs2298383 genotypes by selected variables in childhood epilepsy (CE) patients.

Variables	CE patients (*n* = 105)			CE patients with NC (*n* = 49)			CE patients with DC (*n* = 82)			CE patients with NC/DC (*n* = 36)			*p* value^*a*^	*p* value^*b*^	*p* value^*c*^
				
	CC	CT	TT	CC	CT	TT	CC	CT	TT	CC	CT	TT			
**Confirm age (years)**															
1 month–2	10 (26.3%)	18 (47.4%)	10 (26.3%)	16 (51.6%)	11 (35.5%)	4 (12.9%)	21 (40.4%)	20 (38.4%)	11 (21.2%)	13 (52.0%)	8 (32.0%)	4 (16.0%)	0.083	0.38	0.12
2–16	11 (16.4%)	46 (68.7%)	10 (14.9%)	4 (22.2%)	14 (77.8%)	0	5 (16.7%)	23 (76.7%)	2 (6.6%)	1 (9.1%)	10 (90.9%)	0	0.70a	0.47a	0.96a
**Gender**															
Male	13 (21.3%)	41 (67.2%)	7 (11.5%)	11 (39.3%)	15 (53.6%)	2 (7.1%)	15 (33.3%)	25 (55.6%)	5 (11.1%)	6 (31.6%)	11 (57.9%)	2 (10.5%)	0.2	0.37	0.76a
Female	8 (18.2%)	23 (52.3%)	13 (29.5%)	9 (42.9%)	10 (47.6%)	2 (9.5%)	11 (29.7%)	18 (48.7%)	8 (21.6%)	8 (47.0%)	7 (41.2%)	2 (11.8%)	0.054	0.43	0.27a
**Febrile seizure**															
Yes	7 (25.0%)	16 57.1%)	5 (17.9%)	3 (37.5%)	5 (62.5%)	0	2 (15.4%)	10 (76.9%)	1 (7.7%)	1 (20.0%)	4 (80.0%)	0	0.57b	0.64b	0.57b
No	14 (18.2%)	48 (62.3%)	15 (19.5%)	17 (41.5%)	20 (48.8%)	4 (9.7%)	24 (34.8%)	33 (47.8%)	12 (17.4%)	13 (41.9%)	14 (45.2%)	4 (12.9%)	0.019*	0.07	0.036*
**History of status epilepticus**															
Yes	0	1 (50.0%)	1 (50.0%)	9 (60.0%)	6 (40.0%)	0	6 (54.5%)	4 (36.4%)	1 (9.1%)	6 (75.0%)	2 (25.0%)	0	0.12b	0.29b	0.20b
No	21 (20.4%)	63 (61.2%)	19 (18.4%)	11 (32.3%)	19 (55.9%)	4 (11.8%)	20 (28.2%)	39 (54.9%)	12 (16.9%)	8 (28.6%)	16 (57.1%)	4 (14.3%)	0.31	0.49	0.62
**History of abnormal pregnancy**															
Yes	0	2 (66.7%)	1 (33.3%)	7 (58.3%)	4 (33.4%)	1 (8.3%)	6 (31.6%)	10 (52.6%)	3 (15.8%)	6 (54.5%)	4 (36.4%)	1 (9.1%)	0.37b	0.47b	0.40b
No	21 (20.6%)	62 (60.8%)	19 (18.6%)	13 (35.1%)	21 (56.8%)	3 (8.1%)	20 (31.7%)	33 (52.4%)	10 (15.9%)	8 (32.0%)	14 (56.0%)	3 (12.0%)	0.12	0.27	0.43c
**Abnormal delivery or delayed discharge from NICU**															
Yes	1 (33.3%)	1 (33.3%)	1 (33.3%)	5 (45.4%)	5 (45.5%)	1 (9.1%)	8 (42.1%)	9 (47.4%)	2 (10.5%)	4 (40.0%)	5 (50.0%)	1 (10.0%)	0.40b	0.37b	0.42b
No	20 (19.6%)	63 (61.8%)	19 (18.6%)	15 (39.5%)	20 (52.6%)	3 (7.9%)	18 (28.6%)	34 (54.0%)	11 (17.4%)	10 (38.5%)	13 (50.0%)	3 (11.5%)	0.033*	0.41	0.12
**Family history of epilepsy**															
Yes	4 (21.0%)	11 (57.9%)	4 (21.1%)	5 (100.0%)	0	0	2 (22.2%)	4 (44.5%)	3 (33.3%)	2 (10.0%)	0	0	0.54b	0.65b	—
No	17 (19.8%)	53 (61.6%)	16 (18.6%)	15 (34.1%)	25 (56.8%)	4 (9.1%)	24 (32.9%)	39 (53.4%)	10 (13.7%)	12 (35.3%)	18 (52.9%)	4 (11.8%)	0.12	0.16	0.18

^*a*^*p*-value obtained from CE patients with NC group (*n* = 49) *versus* CE patients group (*n* = 105). ^*b*^*p*-value obtained from CE patients with DC group (*n* = 82) *versus* CE patients group (*n* = 105). ^*c*^*p*-value obtained from CE patients with NC/DC group (*n* = 36) *versus* CE patients group (*n* = 105). abc, comparison of case number between TT and CC + CT genotypes using chi-square with Yate’s correction, Fisher‘s exact test, and Pearson chi-square test, respectively. *p < 0.05 *versus* CE patients group.

### Target Pathway/Function Network of *ADORA2A* in Epilepsy

**FIGURE 1:**
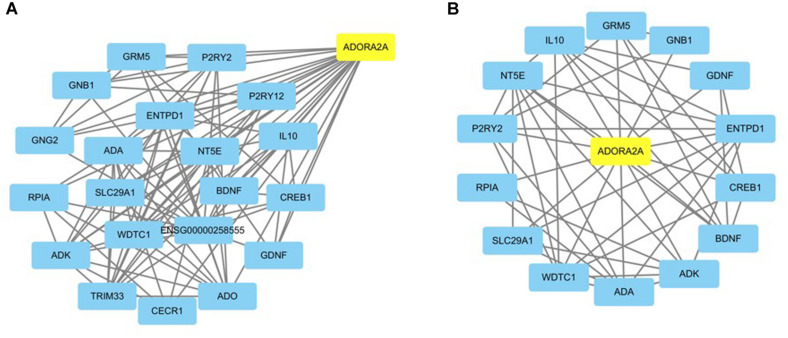
Protein–protein interaction (PPI) network analysis of ADORA2A. **(A)** The PPI network of ADORA2A comprised 21 nodes and 103 edges. **(B)** The PPI network between ADORA2A and 14 common genes associated with epilepsy comprised 15 nodes and 50 edges. Each target protein was represented by a node.

The functional relationships and the interactions for *ADORA2A* were determined in the STRING database. The PPI network comprising 21 nodes and 103 edges was generated (Figure 1A). The results showed that 20 genes interacted with *ADORA2A.* Furthermore, we identified 1,176 epilepsy-associated genes from DisGeNET and 5,064 genes from GeneCards database. After removing the duplicates from two databases, 5,166 genes were finally obtained for epilepsy and then intersected with 20 genes of *ADORA2A* from the PPI analysis. Fourteen common genes including *BDNF*, *GRM5*, *ADK*, *CREB1*, *GNB1*, *IL10*, *ADA*, *ENTPD1*, *WDTC1*, *NT5E*, *GDNF*, *P2RY2*, *SLC29A1*, and *RPIA* were identified as potential targets of *ADORA2A* in epilepsy. *NT5E*, *ENTPD1*, *ADA*, and *ADK* were the top four key genes related to *ADORA2A* according to a high-ranking interaction score. The further PPI network between 14 targets and *ADORA2A* is shown in Figure 1B.

**FIGURE 2:**
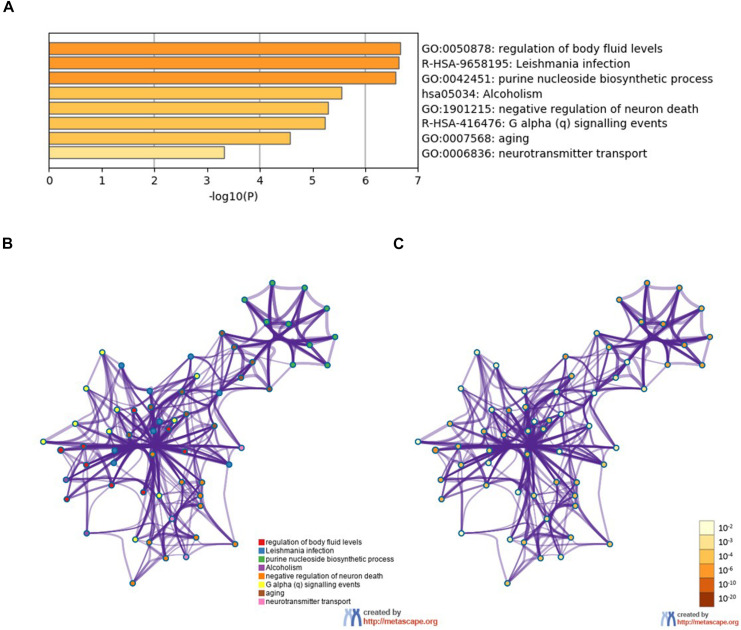
Pathway and process enrichment analysis of the 14 target genes of *ADORA2A*. **(A)** The top non-redundant enrichment clusters by Metascape and statistical significance of one per cluster represented by a discrete color scale. **(B,C)** Metascape visualization of the interactome network. The nodes are colored according to their identities **(B)** and *p*-values **(C)**. Each circle node represents a term, where its size is proportional to the number of input genes falling into that term, and its color represents its cluster identity (nodes of the same color belong to the same cluster). The darker the color, the more statistically significant the node is (see the legend for *p*-value ranges). Terms with a similarity score > 0.3 are linked by an edge (the thickness of the edge represents the similarity score). The network is visualized with Cytoscape (v3.6.0) with “force-directed” layout and with the edge bundled for clarity.

To further analyze the signaling pathways and the functions of *ADORA2A* target genes related to epilepsy, the pathway and process enrichment analysis was performed using Metascape. The results indicated that these 14 genes were mainly involved in the “regulation of body fluid levels,” “leishmania infection,” “purine nucleoside biosynthetic process,” “alcoholism,” “negative regulation of neuron death,” “G alpha (q) signaling events,” “aging,” and “neurotransmitter transport” which were the top eight clusters by *p*-values(Supplementary Table S2, Figure 2). “Negative regulation of neuron death” biological processes contained the most genes (*n* = 9), followed by “G alpha (q) signaling events” and “aging” (Table 5). Four key epilepsy-associated genes for *ADORA2A* principally belong to “purine nucleoside biosynthetic process,” “leishmania infection,” “negative regulation of neuron death,” and “aging,” most of which are concerned with epileptogenesis. These results implied that *ADORA2A* exerted its role in epilepsy in a multiple-target/pathway manner.

**TABLE 5:** Pathway and process enrichment analysis of 14 *ADORA2A* target genes.

Category	Term	Description	Gene
Gene Ontology (GO) biological processes	GO:1901215	Negative regulation of neuron death	BDNF, CREB1, GDNF, IL10, ADA, GRM5, SLC29A1, P2RY2, WDTC1
Reactome gene sets	R-HSA-416476	G alpha (q) signaling events	CREB1, GNB1, GRM5, P2RY2, WDTC1, IL10, ADA
GO biological processes	GO:0007568	Aging	ADA, CREB1, GRM5, IL10, SLC29A1, NT5E, WDTC1
Reactome gene sets	R-HSA-9658195	Leishmania infection	ENTPD1, CREB1, GNB1, IL10, NT5E, WDTC1, P2RY2
GO biological processes	GO:0050878	Regulation of body fluid levels	ADA, ENTPD1, CREB1, SLC29A1, GNB1, P2RY2
GO biological processes	GO:0042451	Purine nucleoside biosynthetic process	ADA, ADK, NT5E, ENTPD1, GDNF, RPIA
Kyoto Encyclopedia of Genes and Genomes pathway	hsa05034	Alcoholism	BDNF, CREB1, SLC29A1, GNB1
GO biological processes	GO:0006836	Neurotransmitter transport	ADA, SLC29A1, GDNF, IL10

## Discussion

In this study, a high incidence rate of epileptic comorbidities was observed in children with epilepsy. There were 24.5% of children with comorbid neurologic disorders and 41% of children with comorbid developmental disorders among 200 CE patients, which was consistent with the reported morbidities (Gaitatzis et al., 2004; Aaberg et al., 2016). Some neurologic disorders of CE, such as epileptic encephalopathy, are usually comorbid severe seizures and the associated intellectual and behavioral disabilities. We found that a younger age of first CE diagnosis, history of status epilepticus, abnormal pregnancy, abnormal delivery, and delayed discharge from NICU might be risk factors of CE comorbid neurologic and developmental disorders.

Epilepsies may derive from genetic abnormalities and well-defined structural and metabolic disorders. It is reported that more than 50% of epilepsies have a genetic basis (Pal et al., 2010). Development in genetic technology contributes to the identification of an increasing number of genes associated with epilepsy (Wang et al., 2017). Hundreds of genes have been found to be associated with epilepsy (Wang et al., 2017). Recently, the role of genetic polymorphism in epileptogenesis and responsiveness of antiepileptic drug has been widely investigated (Löscher et al., 2009; Bertok et al., 2017), which is beneficial to the diagnosis and treatment of epilepsy. The SNP rs2298383 of *ADORA2A* gene located in a potential promoter region upstream of the newly identified exon 1 variants is a functional variant which may affect the rate of gene transcription (Hohoff et al., 2010). Our study is the first to reveal the association between *ADORA2A* rs2298383 variant and CE and its comorbidities. The results indicated that carriers of TT genotype tended to have a lower epilepsy risk for children, and heterozygous CT genotype predisposed children to epilepsy. Furthermore, CC genotype was found to be associated with a higher risk of developing neurologic comorbidity for CE patients compared with TT and CT genotypes. The effect of *ADORA2A* rs2298383 polymorphism on the risk of epilepsy and its comorbidities was similar to other psychopathological diseases. The TT genotype was associated with a decreased risk for current depression and disturbances in sleep and attention (Fraporti et al., 2019; Oliveira et al., 2019), and CC genotype was related to methotrexate-related leukoencephalopathy and levodopa-induced dyskinesia (Tsujimoto et al., 2016; Santos-Lobato et al., 2020). The results of *ADORA2A* rs2298383 polymorphism could not only help to predict the epilepsy risk for healthy individuals and the epileptic comorbidities for CE children but also provide valuable information for clinicians to develop individualized therapy.

Numerous evidences revealed genetic associations between the *ADORA2A* gene and different neurologic and developmental/psychiatric disorders. The genetic polymorphism of *ADORA2A* was found to be associated with encephalopathy with febrile status epilepticus, ADHD traits, and abnormal neurobehavioral performance during sleep restriction (Molero et al., 2013; Rupp et al., 2013; Shinohara et al., 2013; Fraporti et al., 2019). A reduced expression of ADORA2A was observed in the peri-tumor tissue of patients with epilepsy contrary to patients without epilepsy (Huang et al., 2016). Furthermore, dysregulation in ADORA1/ADORA2A expression was associated with glioma development, and a low level of ADORA1/ADORA2A expression could increase the susceptibility of tumor-associated epilepsy (Huang et al., 2016).

BDNF, as a member of the neurotrophic factor, regulates neuronal survival, growth, and connectivity during development and participates in the plasticity and maintenance of neurons throughout adulthood. BDNF binds to its high-affinity receptors (NTRK2) to exert its effects (Nair and Vaidya, 2006). A high amount of BDNF exists in the brain tissue of patients with intractable temporal lobe epilepsy (Binder et al., 2001). Several studies have investigated the association between *BDNF* gene variation and epilepsy (Kanemoto et al., 2003; Xu et al., 2018). BDNF and NTRK2 are found to be correlated with ADORA2A, which are involved in neuroprotective signaling. In our study, *BDNF* was identified as the target gene of ADORA2A on epilepsy by PPI analysis. No significant differences in genotypic distribution and allelic frequencies of the *BDNF* rs6265 and *NTRK2* rs1778929 were observed between controls and cases and between CE patients with and without comorbidities, which were consistent with the reported literature (Kanemoto et al., 2003; Lohoff et al., 2005; Bragatti et al., 2010).

Based on the association study, the underlying mechanisms of *ADORA2A* in epilepsy were further explored by bioinformatics analysis. Fourteen epilepsy-associated genes were identified as potential targets of *ADORA2A*. It suggested that *ADORA2A* might directly regulate or be regulated by these genes to exert its effect on epilepsy. Further functional enrichment analysis indicated that these genes were principally involved in “purine nucleoside biosynthetic process” and “negative regulation of neuron death” biological processes. Neuronal death is found to be associated with the development of epilepsy. Preventing seizure-induced neuronal death in chronic epilepsy and status epilepticus will be a valuable strategy for future seizure management. The purine nucleoside adenosine, as an intercellular signaling molecule, is neuroprotective and plays a neuromodulatory role in the brain. Purinergic signaling involved in cell-to-cell communication through activation of adenosine receptor A2A-R (encoded by ADORA2A), as well as NTRK2 receptors and BDNF, is associated with neuroprotective signaling (Queiroga et al., 2016; Pradhan et al., 2019). Furthermore, purinergic signaling by adenosine and its metabolites is crucial to maintain homeostatic adenosine levels in the brain by regulatory metabolic enzymes including ADK, 5′-NT, and ADA (Jacqueline and Hubbard, 2016). ADA and ADK, identified as key target genes of ADORA2A on epilepsy, are involved in glioma progression, and their upregulated expression in peritumoral tissues is associated with epilepsy in glioma patients (Huang et al., 2015). Additionally, ENTPD1 and NT5E are plasma membrane proteins responsible for hydrolyzing extracellular ATP and ADP to AMP and hydrolyzing AMP to adenosine, respectively. The chromosomal position of human CD39 (ENTPD1)/ecto-apyrase (10g23.1 to q24.1) is collocated with the gene related to partial human epilepsy with audiogenic symptoms (10q.22 to 24) (Maliszewski et al., 1994; Ottman et al., 1995). NT5E regulates the final and the rate-limiting step of adenosine formation controlling immune modulation in the central nervous system (Zimmermann et al., 2012). The changes in nucleotide hydrolysis are regarded as an important mechanism in the modulation of epileptogenesis, and the nucleotidase pathway could regulate the evolution of behavioral and pathophysiological changes related to temporal lobe epilepsy (Schetinger et al., 2007). These suggest that ADORA2A exerted its effects on the genesis of epilepsy and other neurologic disorders possibly through preventing neuron death and regulating purine nucleoside biosynthesis by purinergic signaling.

Due to the relatively small sample size in the present study, further analysis between subgroups with different types of epilepsy is not feasible, and the stratification analysis of *ADORA2A* rs2298383 genotypes in CE patients with and without comorbidities is hard for a meaningful difference to be obtained. Our ongoing study will increase the sample size and validate the role of *ADORA2A*, *BDNF*, and *NTRK2* in epilepsy. The data from bioinformatics analysis lack experimental validation including *in vitro* and *in vivo* studies, which will further be performed to elucidate the underlying mechanisms of *ADORA2A* on epilepsy. Additionally, considering that gene polymorphism may possibly alter gene transcription, *ADORA2A* mRNA expression, ADORA2A protein expression, and intracellular cAMP level in different genotypes of *ADORA2A* rs2298383 will be investigated to provide insights into the molecular mechanisms of ADORA2A on CE and the comorbidities.

## Conclusion

The present study showed that epileptic children with a younger age, history of status epilepticus, abnormal pregnancy, abnormal delivery, or delayed discharge from the NICU tended to suffer neurologic or developmental disorders. Gene polymorphism of *ADORA2A* rs2298383 was associated with CE and its comorbidities. Carriers of *ADORA2A* rs2298383 TT genotype tended to have a lower epilepsy risk in children, while the CT genotype was related to a higher risk. *ADORA2A* rs2298383 CC genotype was predisposed to comorbid neurologic disorders. Fourteen epilepsy-associated genes were identified as potential targets of *ADORA2A* in epilepsy, and four key genes including *NT5E, ENTPD1*, *ADA*, and *ADK* were mainly involved in the “negative regulation of neuron death” and “purine nucleoside biosynthetic process” biological processes. The results suggest that rs2298383 mutation of *ADORA2A* gene deserves attention in CE. Our ongoing study will further validate the role of *ADORA2A* in epilepsy and provide insights into the molecular mechanisms of neurologic and developmental comorbidities.

## Data Availability Statement

The raw data supporting the conclusions of this article will be made available by the authors, without undue reservation.

## Ethics Statement

The studies involving human participants were reviewed and approved by Baoan Women’s and Children’s Hospital Ethics Committee. Written informed consent to participate in this study was provided by the participants’ legal guardian/next of kin.

## Author Contributions

XF, BL, and XX designed the study and wrote the protocol. YC and WL managed the literature searches and collected the clinical data. HX and YY as neurologic clinicians, diagnosed diseases and enrolled the participants of the case–control study. YC, HG, and SX collected the blood samples. WL conducted the bioinformatics analysis. CX undertook the statistical analysis. XF and BL wrote the first draft of the manuscript. All the authors have contributed to and approved the final manuscript.

## Conflict of Interest

The authors declare that the research was conducted in the absence of any commercial or financial relationships that could be construed as a potential conflict of interest.

## Footnotes

1 http://string-db.org

2 http://www.disgenet.org/

3 https://www.genecards.org/

4 http://metascape.org

## Supplementary Material

Supplementary Table 1 The information of variants investigated in this study.

Supplementary Table 2 Functional enrichment analysis of fourteen *ADORA2A* target genes.

Supplementary Table 3 The ADORA2A rs2298383 genotype of healthy children and CE patients.

## Abbreviations

ADA: adenosine deaminase
ADHD: attention deficit–hyperactivity disorder
ADK: adenosine kinase
ADORA1: adenosine A1 receptor
ADORA2A: adenosine A2A receptor
AESD: acute encephalopathy with biphasic seizures and late reduced diffusion
BDNF: brain-derived neurotrophic factor
CE: childhood epilepsy
CI: confidence interval
CREB1: cAMP responsive element binding protein 1
DC: developmental comorbidity
ENTPD1: ectonucleoside triphosphate diphosphohydrolase 1
GDNF: glial cell line derived neurotrophic factor
GNB1: G protein subunit beta 1
GRM5: glutamate metabotropic receptor 5
IDD: developmental disabilities
IL10: interleukin 10
NC: neurologic comorbidity
NT5E: 5’-nucleotidase ecto
NTRK2: neurotrophic receptor tyrosine kinase 2
OR: odds ratio
P2RY2: purinergic receptor P2Y2
PPI: protein–protein interaction
RPIA: ribose 5-phosphate isomerase A
SLC29A1: solute carrier family 29 member 1
SNP: single nucleotide polymorphism
WDTC1: WD and tetratricopeptide repeats 1
5’-NT: 5’-nucleotidase.
