# Supplementary material for: Genetic Polymorphism of ADORA2A Is Associated With the Risk of Epilepsy and Predisposition to Neurologic Comorbidity in Chinese Southern Children
Source: Front Neurosci. 2020 Nov 11;14:590605. doi: 10.3389/fnins.2020.590605 (PMC7686584; doi:10.3389/fnins.2020.590605)
Supplement: Supplementary Table 1 — The information of variants investigated in this study. [file Table_1.pdf]

Supplementary table 1. The information of variants investigated in this study.

Gene	SNP	Alleles	Position	Variation Type	Functional Consequence
ADORA2A	rs2298383	C>A,G,T	chr22:24429543 (GRCh38.p12)	single nucleotide variation	Intron variant, genic downstream transcript variant, non-coding transcript variant
BDNF	rs6265	C>T	chr11:27658369 (GRCh38.p12)	single nucleotide variation	Missense variant, non-coding transcript variant, coding sequence variant
NTRK2	rs1778929	T>C	chr9:84707625 (GRCh38.p12)	single nucleotide variation	Intron Variant

Supplementary table 2. Functional enrichment analysis of fourteen *ADORA2A* target genes

Category	Term	Description	LogP	Log(q-value)	Genes
GO Biological Processes	GO:0050878	Regulation of body fluid levels	-6.665	-3.013	ADA, ENTPD1, CREB1, SLC29A1, GNB1, P2RY2
Reactome Gene Sets	R-HSA-9658195	Leishmania infection	-6.632	-3.013	ENTPD1, CREB1, GNB1, IL10, NT5E, WDTC1, P2RY2
GO Biological Processes	GO:0042451	Purine nucleoside biosynthetic process	-6.565	-3.013	ADA, ADK, NT5E, ENTPD1, GDNF, RPIA
KEGG Pathway	hsa05034	Alcoholism	-5.550	-2.273	BDNF, CREB1, SLC29A1,GNB1
GO Biological Processes	GO:1901215	Negative regulation of neuron death	-5.285	-2.080	BDNF, CREB1, GDNF, IL10, ADA, GRM5, SLC29A1, P2RY2, WDTC1
Reactome Gene Sets	R-HSA-416476	G alpha (q) signalling events	-5.236	-2.063	CREB1, GNB1, GRM5, P2RY2, WDTC1, IL10, ADA
GO Biological Processes	GO:0007568	Aging	-4.570	-1.613	ADA, CREB1, GRM5, IL10, SLC29A1, NT5E, WDTC1
GO Biological Processes	GO:0006836	Neurotransmitter transport	-3.324	-0.783	ADA, SLC29A1, GDNF, IL10
