# Supplementary material for: Genetic Polymorphism of ADORA2A Is Associated With the Risk of Epilepsy and Predisposition to Neurologic Comorbidity in Chinese Southern Children
Source: Front Neurosci. 2020 Nov 11;14:590605. doi: 10.3389/fnins.2020.590605 (PMC7686584; doi:10.3389/fnins.2020.590605)
Supplement: Supplementary Table 3 — The ADORA2A rs2298383 genotype of healthy children and CE patients. [file Data_Sheet_1.pdf]

Supplementary data

Table 3. The ADORA2A rs2298383 genotype of healthy children and CE patients in this study.

Control group (n = 244)		Case group (n = 200)			
No.	Genotype	No.	Genotype	NC	DC
1	CT	1	CT		
2	CC	2	CT		
3	TT	3	CC		
4	CT	4	CT		
5	TT	5	CT		
6	CT	6	CC		
7	CT	7	TT		
8	CT	8	CT		
9	TT	9	CT		
10	TT	10	TT		
11	CT	11	CT		
12	CT	12	CC		
13	CT	13	CT		
14	TT	14	CT		
15	CC	15	CT		
16	CT	16	CC		
17	CC	17	TT		
18	CT	18	CT		
19	TT	19	CT		
20	TT	20	CT		
21	TT	21	CT		
22	TT	22	TT		
23	TT	23	CC		
24	CC	24	TT		
25	TT	25	CT		
26	CT	26	CC		
27	CC	27	CT		
28	CT	28	CC		
29	TT	29	TT		
30	TT	30	TT		
31	CC	31	CT		
32	TT	32	CT		
33	CT	33	CT		
34	TT	34	CT		
35	TT	35	CC		
36	TT	36	CT		

Control group (n = 244)		Case group (n = 200)			
No.	Genotype	No.	Genotype	NC	DC
37	CC	37	CT		
38	CT	38	CT		
39	TT	39	CT		
40	TT	40	CT		
41	TT	41	CT		
42	CT	42	TT		
43	CC	43	CT		
44	CC	44	CT		
45	CC	45	CT		
46	CC	46	CT		
47	TT	47	CT		
48	CC	48	TT		
49	CC	49	CC		
50	CT	50	CT		
51	CC	51	CT		
52	CT	52	TT		
53	CT	53	CC		
54	CT	54	TT		
55	CT	55	CT		
56	CC	56	CC		
57	TT	57	CT		
58	CC	58	CT		
59	CT	59	CT		
60	CT	60	CC		
61	CT	61	TT		
62	TT	62	CT		
63	TT	63	CC		
64	CT	64	CC		
65	CC	65	CT		
66	CT	66	CT		
67	TT	67	CT		
68	CT	68	CT		
69	TT	69	TT		
70	TT	70	CC		
71	CC	71	CT		
72	CT	72	CT		
73	TT	73	CC		
74	CT	74	TT		
75	CC	75	CT		
76	CT	76	CT		
77	TT	77	CT		
78	CC	78	CT		

Control group (n = 244)		Case group (n = 200)			
No.	Genotype	No.	Genotype	NC	DC
79	TT	79	CT		
80	CT	80	CC		
81	CT	81	CT		
82	CT	82	CT		
83	TT	83	CT		
84	CT	84	CT		
85	CT	85	CT		
86	CC	86	CT		
87	CT	87	CT		
88	CC	88	CC		
89	CT	89	CT		
90	CT	90	TT		
91	CT	91	CC		
92	TT	92	CT		
93	CT	93	CC		
94	CT	94	TT		
95	CT	95	TT		
96	TT	96	CC		
97	CT	97	TT		
98	CC	98	CT		
99	CT	99	CT		
100	CC	100	CT		
101	TT	101	CT		
102	CT	102	CT		
103	TT	103	CT		
104	CT	104	TT		
105	CT	105	TT		
106	CC	106	CT	√	√
107	TT	107	CT	√	√
108	CT	108	CC	√	√
109	CT	109	CC	√	√
110	TT	110	CT	√	√
111	CT	111	CC	√	√
112	TT	112	CC	√	√
113	CC	113	CC	√	√
114	CT	114	CC	√	√
115	TT	115	CT	√	√
116	TT	116	CT	√	√
117	CT	117	CT	√	√
118	TT	118	CT	√	√
119	TT	119	TT	√	√
120	CC	120	TT	√	√

Control group (n = 244)		Case group (n = 200)			
No.	Genotype	No.	Genotype	NC	DC
121	CC	121	CC	√	√
122	CT	122	CT	√	√
123	TT	123	CC	√	√
124	TT	124	CT	√	√
125	CC	125	CC	√	√
126	CC	126	CC	√	√
127	CT	127	CT	√	√
128	CT	128	CC	√	√
129	CT	129	TT	√	√
130	CC	130	CC	√	√
131	CC	131	CT	√	√
132	CC	132	TT	√	√
133	CC	133	CC	√	√
134	CT	134	CT	√	√
135	CC	135	CT	√	√
136	CT	136	CT	√	√
137	CT	137	CT	√	√
138	TT	138	CT	√	√
139	TT	139	CT	√	√
140	CT	140	CC	√	√
141	CC	141	CT	√	√
142	CT	142	CC		√
143	CT	143	CC		√
144	CT	144	CC		√
145	CC	145	CT		√
146	CT	146	TT		√
147	TT	147	CT		√
148	CC	148	TT		√
149	CT	149	TT		√
150	TT	150	CT		√
151	CT	151	CT		√
152	CT	152	TT		√
153	CT	153	CT		√
154	CC	154	CC		√
155	CC	155	TT		√
156	CT	156	CT		√
157	CC	157	TT		√
158	CT	158	CT		√
159	CT	159	CT		√
160	TT	160	CT		√
161	CT	161	CT		√
162	CT	162	CT		√

Control group (n = 244)		Case group (n = 200)			
No.	Genotype	No.	Genotype	NC	DC
163	CC	163	CC		√
164	TT	164	CT		√
165	CC	165	CT		√
166	TT	166	CT		√
167	CC	167	CT		√
168	TT	168	CC		√
169	CC	169	CT		√
170	CT	170	TT		√
171	CC	171	CC		√
172	CT	172	TT		√
173	CC	173	CT		√
174	CT	174	CT		√
175	CT	175	CC		√
176	CC	176	CT		√
177	CT	177	CT		√
178	CT	178	CC		√
179	CT	179	CT		√
180	CT	180	TT		√
181	CT	181	CC		√
182	CT	182	CT		√
183	CC	183	CT		√
184	CT	184	CT		√
185	CC	185	CC		√
186	TT	186	CT		√
187	CT	187	CC		√
188	CT	188	CT	√	
189	CT	189	CC	√	
190	CT	190	CC	√	
191	CT	191	CT	√	
192	CT	192	CT	√	
193	CC	193	CC	√	
194	TT	194	CT	√	
195	CC	195	CT	√	
196	TT	196	CC	√	
197	CC	197	CT	√	
198	TT	198	CC	√	
199	TT	199	CT	√	
200	TT	200	CC	√	
201	CC				
202	CT				
203	CC				
204	TT				

Control group (n = 244)		Case group (n = 200)			
No.	Genotype	No.	Genotype	NC	DC
205	CT				
206	CC				
207	CT				
208	CT				
209	CC				
210	CT				
211	CT				
212	TT				
213	TT				
214	TT				
215	CT				
216	CT				
217	CT				
218	TT				
219	TT				
220	TT				
221	CT				
222	CT				
223	TT				
224	TT				
225	CT				
226	TT				
227	TT				
228	CC				
229	CC				
230	CT				
231	CT				
232	CT				
233	CT				
234	CC				
235	CT				
236	CT				
237	TT				
238	CC				
239	TT				
240	CT				
241	CT				
242	CT				
243	CT				
244	CT				

NC, neurologic comorbidity; DC, developmental comorbidity.
